# Preferentially expressed antigen in melanoma as a novel diagnostic marker differentiating thymic squamous cell carcinoma from thymoma

**DOI:** 10.1038/s41598-020-69260-z

**Published:** 2020-07-23

**Authors:** Yohei Taniguchi, Mitsuaki Ishida, Tomohito Saito, Hironori Ryota, Takahiro Utsumi, Natsumi Maru, Hiroshi Matsui, Haruaki Hino, Koji Tsuta, Tomohiro Murakawa

**Affiliations:** 1 Department of Thoracic Surgery, Kansai Medical University, 2-5-1 Shinmachi, Hirakata, Osaka 573-1010 Japan; 2 Department of Pathology and Laboratory Medicine, Kansai Medical University, 2-5-1 Shinmachi, Hirakata, Osaka 573-1010 Japan; 3 Department of Surgery, Kansai Medical University, 2-5-1 Shinmachi, Hirakata, Osaka 573-1010 Japan

**Keywords:** Biomarkers, Oncology

## Abstract

Thymic squamous cell carcinoma (TSQCC), accounting for 70–80% of thymic carcinoma cases, is distinct from thymoma. However, differential diagnosis for type B3 thymoma is sometimes challenging, even with established markers for TSQCC, including KIT and CD5, which are expressed in ~ 80% of TSQCCs and ~ 3% of thymomas. Novel TSQCC-specific markers would facilitate precise diagnosis and optimal treatment. Herein, we found that preferentially expressed antigen in melanoma (PRAME) may be a novel TSQCC-specific diagnostic marker. We comprehensively profiled 770 immune-related mRNAs in 10 patients with TSQCC and two healthy controls, showing that *PRAME* and *KIT* were significantly upregulated in TSQCC (adjusted *p* values = 0.045 and 0.0011, respectively). We then examined PRAME expression in 17 TSQCCs and 116 thymomas via immunohistochemistry. All 17 (100%) TSQCCs displayed diffuse and strong PRAME expression, whereas eight of 116 (6.8%) thymomas displayed focal and weak expression (*p* < 0.0001). KIT and CD5 were positive in 17 (100%) and 16 (94.1%) TSQCCs, respectively, whereas one (0.9%) type B3 thymoma showed double positivity for KIT and CD5. The KIT-/CD5-positive type B3 thymoma was negative for PRAME. Thus, combinatorial evaluation of PRAME with KIT and CD5 may facilitate a more precise diagnosis of TSQCC.

## Introduction

Thymic epithelial tumours are rare malignancies, but are the most frequent type of anterior mediastinal malignancy, with an incidence rate of 1–7 individuals/million population/year^1–3^. According to the Japanese Association for Research of the Thymus database, most (~ 87%) thymic epithelial tumours are comprised of thymoma, followed by thymic carcinoma (~ 11%) and neuroendocrine tumour (~ 2%)^4^. Thymic squamous cell carcinoma (TSQCC), accounting for 70–80% of thymic carcinoma cases, shows clinical and pathological distinctions from thymoma. TSQCC shows an aggressive clinical course, developing nodal and distant metastasis with pathological features of squamous cell carcinoma lacking immature T lymphocytes. In contrast, thymoma shows relatively slow progression with local invasion, exhibiting organotypic (thymus-like) features. Therefore, histopathological diagnosis of TSQCC is usually straightforward, but differential diagnosis from type B3 thymoma is challenging in some cases^2^.

KIT (also known as CD117) and CD5 are established diagnostic markers for TSQCC and are almost always negative in thymoma, with some exceptions^1,5–8^. Briefly, KIT and CD5 are positive in ~ 80% of TSQCCs, whereas ~ 3% of thymomas show positivity for KIT or CD5^1,4^. Other markers, such as glucose transporter 1 and MUC1, have been proposed as TSQCC markers (positive in ~ 90% of cases); however, the positive rate in type B3 thymoma is relatively high (~ 40% and ~ 10%, respectively)^1,9^. Identification of a novel marker specific to TSQCC will facilitate precise diagnosis and lead to optimal management of patients with thymic epithelial malignancy.

Accordingly, in this study, we identified preferentially expressed antigen in melanoma (PRAME), a type of cancer-testis antigen, as a novel diagnostic marker of TSQCC for the first time, based on comprehensive mRNA expression analysis and immunohistochemical validation.

## Results

### Clinical and pathological characteristics of the study population

The median ages of patients with TSQCC and thymoma were 64 years (range 46–80 years) and 62 years (range 24–86 years), respectively. There were seven women (41.1%) in the TSQCC group and 65 women (57.0%) in the thymoma group. Among the 116 patients with thymoma, 12 patients had type A thymomas, 34 had type AB thymomas, 23 had type B1 thymomas, 28 had type B2 thymomas, 17 had type B3 thymomas, and two had micronodular thymomas. No patients received neoadjuvant chemotherapy, radiotherapy, or chemoradiotherapy before surgical resection of TSQCC or thymoma.

### RNA expression profiles of TSQCC

**Figure 1:**
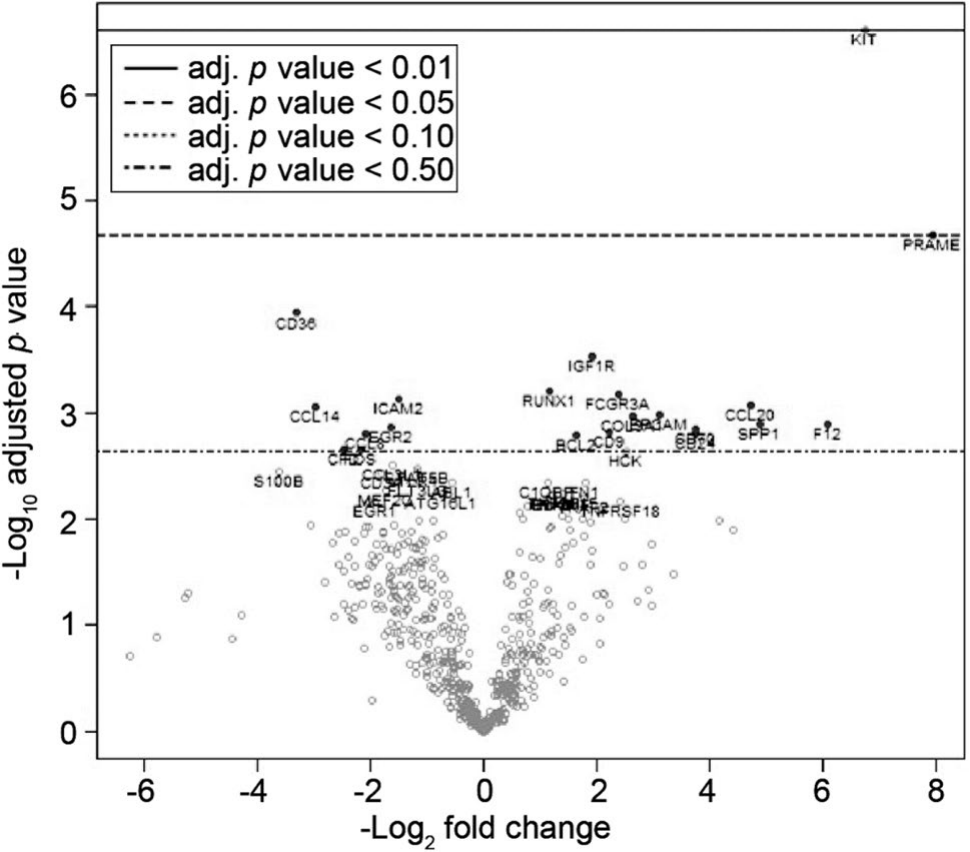
Volcano plot showing differentially expressed genes between thymic squamous cell carcinoma and normal controls. Log_2_ fold change and − log_10_ of adjusted *p* values are plotted on the x- and y-axes, respectively. PRAME, preferentially expressed antigen in melanoma.

Differential gene expression between TSQCC and normal thymus tissues is summarised in the volcano plot shown in Fig. 1, and the top 10 genes differentially expressed in TSQCC compared with normal thymus tissues are summarised in Table 1. *PRAME* and *KIT* showed significant upregulation in TSQCC (adjusted *p* value = 0.045 and 0.0011, respectively).

**Table 1:** Top 10 genes that were differentially expressed in thymic squamous cell carcinoma compared with normal controls according to *p* values.

	Log_2_ (fold change)	SE (log_2_)	*p* value	Adjusted *p* value
*KIT*	6.76	0.50	2.51 × 10^–7^	0.0011
*PRAME*	7.94	0.99	2.15 × 10^–5^	0.045
*CD36*	− 3.31	0.51	0.00012	0.16
*IGF1R*	1.92	0.34	0.00029	0.31
*RUNX1*	1.17	0.23	0.00062	0.36
*FCGR3A*	2.39	0.47	0.00068	0.36
*ICAM2*	− 1.5	0.30	0.00073	0.36
*CCL20*	4.73	0.97	0.00085	0.36
*CCL14*	− 2.98	0.61	0.00087	0.36
*EPCAM*	3.12	0.66	0.0010	0.36

*CCL*, C–C motif chemokine ligand; *EPCAM*, epithelial cell adhesion molecule; *FCGR3A*, Fc gamma receptor 3A; *ICAM2*, intercellular adhesion molecule; *IGF1R*, insulin-like growth factor 1 receptor; *PRAME*, preferentially expressed antigen in melanoma; *RUNX*, Runt-related transcription factor.

### Immunohistochemical profiles of TSQCC and thymoma

**Figure 2:**
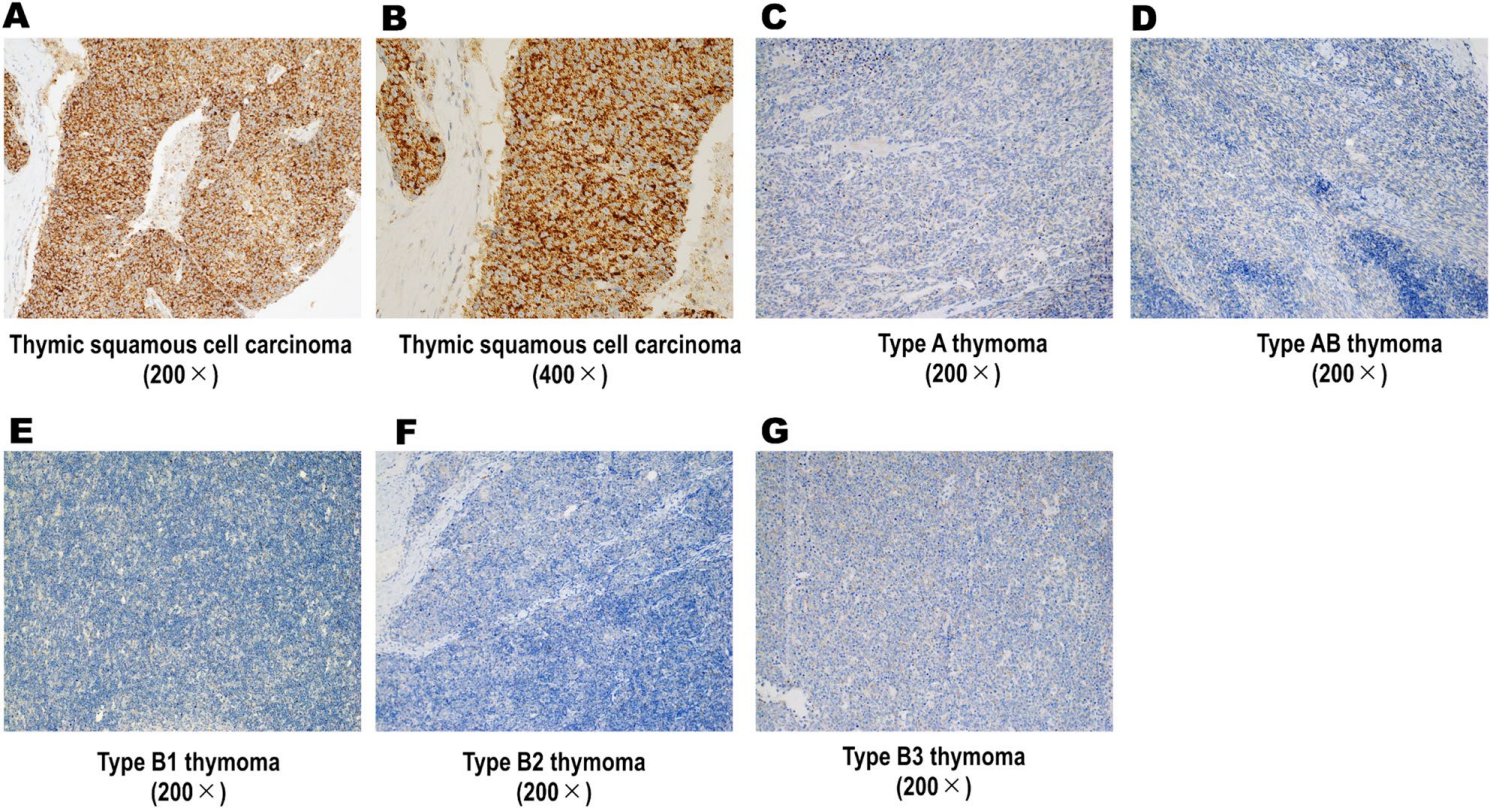
Representative images of PRAME immunohistochemistry staining. (**A**, **B**) Thymic squamous cell carcinoma showed diffuse and strong expression of PRAME. (**C**–**F**) All types of thymomas showed negativity for PRAME with few exceptions. PRAME, preferentially expressed antigen in melanoma.

**Figure 3:**
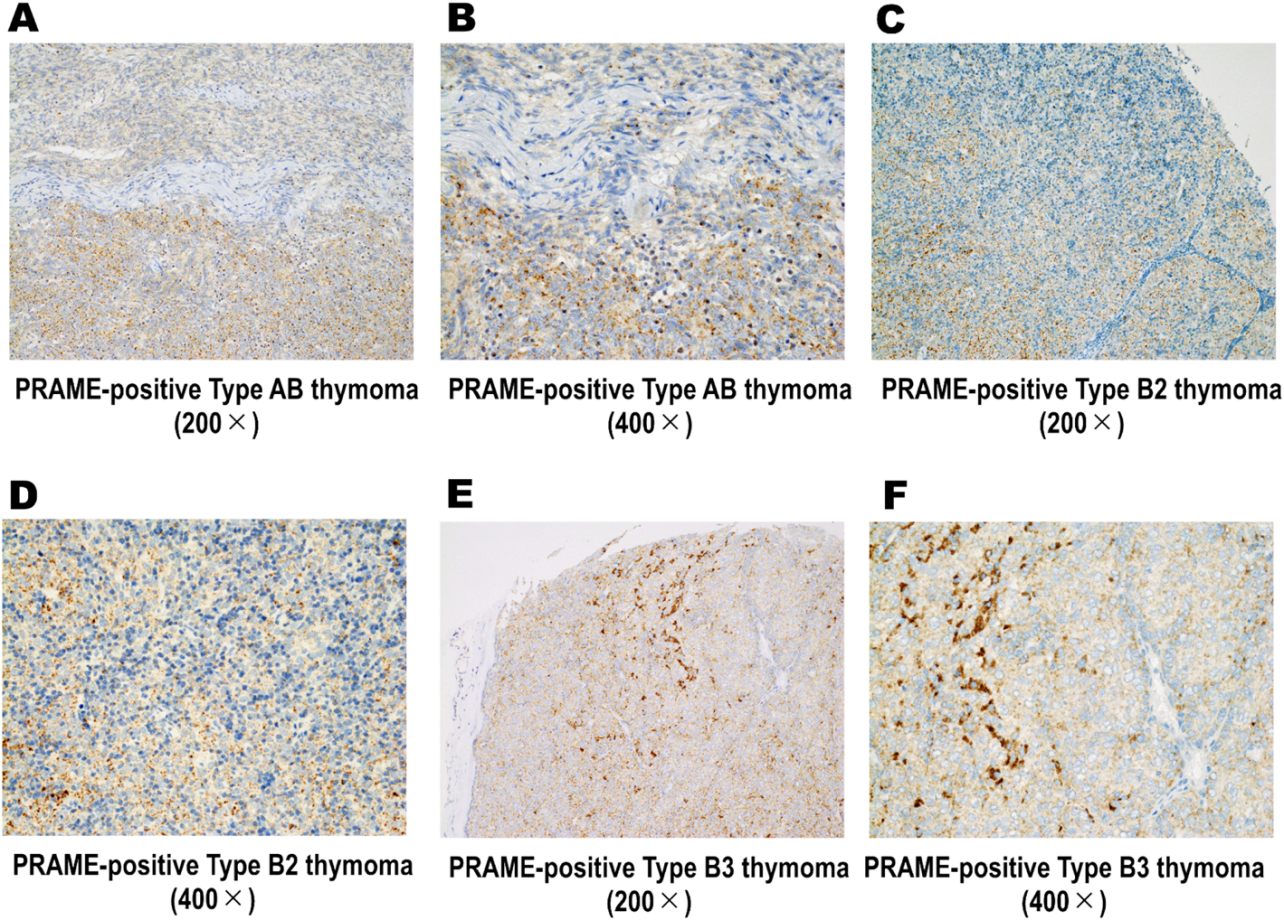
Representative images of PRAME-positive thymomas. (**A**–**F**) Few type AB, B2, and B3 thymomas showed positive staining for PRAME, but the expression pattern was focal and weak.

The results of immunohistochemical analyses are shown in Table 2. PRAME was expressed in all 17 (100%) TSQCCs, whereas eight (6.5%) thymomas and none of the three normal controls showed positivity (*p* < 0.0001, each). Positivity for PRAME was significantly frequent in TSQCCs compared with that in all types (i.e., types A, AB, B1, B2, and B3) of thymoma (*p* < 0.0001, each). Representative images of PRAME immunohistochemistry staining are shown in Fig. 2. Diffuse and strong expression of PRAME was observed in TSQCC, which was distinct from that in thymoma (Fig. 2A–G). This unique immunostaining pattern of TSQCCs was also observed in whole sections. Five of 34 (14.7%) type AB, one of 28 (3.5%) type B2, and two of 17 (11.8%) type B3 thymomas showed positive staining for PRAME. However, the expression pattern was focal and weak (Fig. 3), as validated with whole sections. KIT and CD5 were positive in 17 (100%) and 16 (94.1%) TSQCCs, whereas one (0.9%) type B3 thymoma showed double positivity for KIT and CD5. The KIT-/CD5-positive type B3 thymoma was negative for PRAME. Immunophenotypic patterns of type B3 thymoma are summarised in Table 3. No cases of type B3 thymoma showed simultaneous positivity for PRAME, KIT, and CD5. Patients’ characteristics for PRAME-positive thymoma are summarised in Table 4. There were no significant differences in recurrence-free survival between PRAME-positive type AB, B2, and B3 thymomas and their negative counterparts (type AB, *p* = 1.00; type B2, *p* = 0.735; type B3, *p* = 0.569). No significant differences in presentation stage were found between PRAME-positive and PRAME-negative thymomas (type AB, *p* = 0.954; type B2, *p* = 0.700; type B3, *p* = 0.157). Furthermore, metastatic lesions of PRAME-positive thymomas (n = 4) showed no expression of PRAME, in contrast to metastatic lesions of TSQCC (n = 1), which showed strong and diffuse expression of PRAME.

**Table 2:** Immunophenotypic profiles of thymic squamous cell carcinomas and thymomas.

Histology	n (total)	PRAME-positive n (%)	KIT-positive n (%)	CD5-positive n (%)
TSQCC	17	17 (100%)	17 (100%)	16 (94%)
Thymoma				
Type A	12	0 (0%)	0 (0%)	0 (0%)
Type AB	34	5* (14.7%)	0 (0%)	0 (0%)
Type B1	23	0 (0%)	0 (0%)	0 (0%)
Type B2	28	1* (3.5%)	0 (0%)	0 (0%)
Type B3	17	2* (11.8%)	1 (5.8%)	1 (5.8%)
Micronodular	2	0 (0%)	0 (0%)	0 (0%)
All types	116	8* (6.8%)	1 (0.9%)	1 (0.9%)
Normal control	3	0 (0%)	0 (0%)	0 (0%)

PRAME, preferentially expressed antigen in melanoma; TSQCC, thymic squamous cell carcinoma.

*The expression pattern of PRAME in type AB, B2s and B3 thymomas was focal and weak.

**Table 3:** Immunophenotypic patterns of type B3 thymomas (n = 17).

n (%)	PRAME	KIT	CD5
14 (82.3%)	−	−	−
2 (11.7%)	+	−	−
1 (5.8%)	−	+	+

*PRAME*, preferentially expressed antigen in melanoma.

**Table 4:** Patient characteristics for PRAME-positive thymomas.

Patient no	Sex	Age, years	WHO classification	pStage	Postoperative recurrence
PP-Ty 1	F	55	B3	2	None
PP-Ty 2	F	50	AB	1	None
PP-Ty 3	M	64	AB	1	None
PP-Ty 4	F	57	B3	4	None
PP-Ty 5	M	80	AB	2	None
PP-Ty 6	M	81	A2	2	None
PP-Ty 7	F	64	AB	2	None
PP-Ty 8	M	61	AB	2	None

Stage was determined using the 8th TNM staging for thymic epithelial tumours.

### *PRAME* mRNA expression levels in TSQCCs, PRAME-negative thymomas, and PRAME-positive thymomas

*PRAME* mRNA expression in TSQCC was higher than that in PRAME-negative thymomas, with a log_2_ fold change of 3.96 (standard error, 1.18; adjusted *p* value = 0.0498). In contrast, *PRAME* mRNA expression in TSQCCs tended to be higher than that in PRAME-positive thymomas, although the difference did not reach statistical significance (log_2_ fold change, 1.24; standard error, 0.63; adjusted *p* value = 0.911).

## Discussion

Although several studies have analysed the genomic profiles of TSQCC by focusing on mutational status^10–14^, comprehensive mRNA profiling of TSQCC had not been performed previously. However, in this study, we clearly demonstrated the upregulation of *PRAME* mRNA in TSQCCs compared with that in normal control samples and the overexpression of PRAME protein in TSQCCs compared with that in thymomas and normal control samples. To the best of our knowledge, this is the first report identifying PRAME as a novel diagnostic marker for TSQCC, but not for thymoma.

PRAME, a type of cancer-testis antigen, was first recognised as a tumour-associated antigen through analyses of cytotoxic T-cell clones collected from a patient with metastatic malignant melanoma^15^. PRAME can bind to the retinoic acid receptor in the presence of retinoic acid, leading to inhibition of retinoic acid receptor signalling and tumour necrosis factor-related apoptosis-inducing ligand expression. This in turn blocks cell differentiation and promotes cell proliferation by downregulating pro-apoptotic genes^16,17^. In normal tissues, PRAME expression is regulated at very low levels by DNA methylation, except in the testes^15^. Notably, overexpression of PRAME has been observed in malignant melanoma (88% of primary lesions and 95% of metastases), lung carcinoma (46% of adenocarcinomas and 78% of squamous cell carcinomas), renal cell carcinoma, neuroblastoma, myxoid liposarcoma, and synovial sarcoma^15,18–22^. Moreover, PRAME expression correlates with higher tumour grade and poorer prognosis in neuroblastoma, urothelial carcinoma, osteosarcoma, head and neck carcinoma, breast cancer, liposarcoma, chronic myeloid leukaemia, and lymphomas^21–23^.

In this study, we demonstrated the distinct expression patterns of PRAME in TSQCC and thymoma. Given that PRAME inhibits cell differentiation and is expressed in tumour cells with stem cell characteristics^24^, PRAME positivity in TSQCC may reflect the biological distinctions in the differentiation state of the tumour cells compared with thymoma. The underlying mechanisms of PRAME overexpression in TSQCC remain unclear. To date, several mechanisms have been shown to upregulate PRAME in other malignancies, including hypomethylation of 5′-C-phosphate-G-3′ sites in the 3′ sequence of the promoter region and the exon 1b region of *PRAME*, *microRNA-211* downregulation, production of breakpoint cluster region-Abelson murine leukaemia viral oncogene homologue fusion protein, and activation of FMS-like tyrosine kinase 3^23,25–28^.

Importantly, all TSQCCs showed diffuse and strong expression of PRAME, whereas thymomas showed no expression or only focal and weak expression. This indicated that PRAME could be a novel diagnostic marker for TSQCCs. Although some cases of types AB, B2, and B3 thymomas (14.7%, 3.5%, and 11.8%, respectively, in this study) showed positivity for PRAME, the staining pattern was focal and weak. In addition, PRAME-positive thymomas are always KIT- and CD5-negative. Interestingly, a case of type B3 positive for both KIT and CD5 showed no expression of PRAME. These findings suggested that the use of PRAME in combination with KIT and CD5 may facilitate more precise differential diagnosis between TSQCC and thymoma, specifically for type B3 tumours.

From a therapeutic perspective, PRAME has been studied as an attractive target for antigen-specific immunization by adoptive T-cell therapy in some tumours, such as uveal melanoma and non-small cell lung cancer^29–31^. Notably, overexpression of PRAME has been shown to be associated with resistance to chemotherapy^32–34^. Additionally, PRAME may contribute to chemoresistance in TSQCC. The chemosensitivity of thymic carcinoma to classical cytotoxic antitumor agents appears to be heterogenous, varying from unexpected responses to clear nonresponses. Recently, specific therapeutic agents targeting KIT, such as sunitinib and imatinib, have been investigated as potential treatment options; of these, sunitinib appears to be more promising^35^, whereas imatinib failed to show significant therapeutic effect in a clinical trial^36^. Accordingly, therapeutic strategies targeting PRAME could be promising for patients with TSQCC because no standard systemic therapies have been established for this unresectable disease. In our study population, no patients with PRAME-positive thymoma developed postoperative recurrence; thus, we could not examine whether PRAME-positivity affected the chemosensitivity of thymomas.

There were some limitations to this study. First, the presented results were based on a single-centre study with a small sample size. Thus, the findings of the study may not be generalised, and type II error may be present. Further studies are needed to validate our results in larger study populations. Second, PRAME was studied exclusively in TSQCC but not in other histological types of thymic carcinoma. Although TSQCC is a major subtype of thymic carcinoma accounting for ~ 70% of cases, there are 10 other histological variants according to the 4th edition of the World Health Organization classification^2^. Thus, it remains unclear whether overexpression of PRAME is TSQCC-specific or thymic carcinoma-specific. Accordingly, immunohistochemical assessment of other subtypes of thymic carcinoma is urgently needed.

In conclusion, our results suggested that PRAME may be a novel diagnostic marker differentiating TSQCC from thymoma. Specifically, the use of PRAME in combination with KIT and CD5 will facilitate more precise diagnosis of patients with TSQCC. Further investigations are necessary for optimal diagnosis of thymic epithelial malignancy and to improve our understanding of the underlying biology.

## Materials and methods

This study was conducted in accordance with the Declaration of Helsinki and was approved by the Kansai Medical University Hospital Clinical Research Ethics Board (approval nos.: 2015630 and 2017057). Informed consent was obtained from patients by opt-out methodology owing to the retrospective design of the study, with no risk for the participants^37^. Information regarding this study, such as the inclusion criteria and the opportunity to opt out, was provided through the institutional website.

### Case selection

Chart review was carried out for all patients (n = 150) who underwent surgery for thymic epithelial malignancy between January 1, 2006 and December 31, 2019. The inclusion criteria for this study were a histopathological diagnosis of TSQCC or thymoma and adequacy of the surgical specimen for analysis. Nineteen of 150 patients were excluded owing to inadequate sample amount. Two patients had synchronous multiple thymomas: each patient had two thymomas with different WHO types. Finally, 17 patients with TSQCCs and 116 with thymomas were included. None of the 17 patients with TSQCC had previously presented with squamous cell carcinoma that originated from any tissue other than the thymus. For controls, three patients with thymic cysts were included, and non-cystic sections of resected specimens (or normal thymic tissue) from these patients were used for RNA extraction and immunohistochemistry staining.

### Histopathological evaluation

The diagnosis and subclassification of thymoma and the diagnosis of TSQCC were confirmed by two diagnostic pathologists (MI and KT) independently according to the 4th edition of WHO classification^2^.

### RNA extraction

We extracted RNA from the archived samples as described in our previous reports^38^. Briefly, for mRNA extraction, 5-μm-thick sections from formalin-fixed and paraffin-embedded (FFPE) blocks from 10 TSQCCs and two normal controls were cut. RNA was extracted using a NucleoSpin total RNA FFPE kit (Macherey–Nagel GmBH & Co. KG, Düren, Germany), including on-column treatment with DNase. A quantitative evaluation of RNA was performed using a Nanodrop 1,000 spectrophotometer (Thermo Fisher Scientific, Wilmington, DE, USA). RNA quality was evaluated by measuring the 260/280 nm ratio. We excluded samples in which the total amount of RNA was less than 50 ng/μL or the 260/280 ratio was less than 1.6.

### Comprehensive mRNA expression profiling by digital mRNA counts

We performed comprehensive mRNA expression profiling of 770 immune-related genes in 10 patients with TSQCC and two normal controls using the nCounter PanCancer Immune Profiling Panel (NanoString Technologies, Inc., Seattle, WA, USA), according to the manufacturer’s instructions. The RNA was hybridised with the probe sets for 16 h at 67 °C, and the samples were then processed using an automated nCounter Sample Prep Station (NanoString Technologies, Inc.). Cartridges containing immobilised and aligned reporter complexes were subsequently imaged on an nCounter Digital Analyzer (NanoString Technologies, Inc.) that had been set at a data resolution of 555 fields of view. Reporter counts were determined, log_2_-transformed, and normalised using housekeeping genes selected using the nSolver analysis software version 4.0 (NanoString Technologies, Inc.). Fold changes, *p* values, and adjusted *p* values were determined using the nSolver analysis software version 4.0 with nCounter Advanced Analysis add-on software version 2.0.115. In particular, adjusted *p* values were determined using the Benjamini–Yekutieli method to control the false discovery rate^39^.

### Tissue microarrays

Tissue microarrays were created as previously described^40^. Briefly, the most morphologically representative tumour regions were selected using haematoxylin and eosin-stained slides, and two tissue cores (2 mm in diameter) were punched out from the selected regions from the paraffin-embedded blocks for each patient. These tissue cores were then arrayed in a paraffin block. Moreover, two tissue cores from thymic cysts of three patients were also arrayed in a paraffin block.

### Immunohistochemical evaluation and statistical analysis

Immunohistochemical analyses were performed using autostainers (Discovery, Roche Diagnostics, Basel, Switzerland; Dako Autostainer link 48, Agilent Technologies, Santa Clara, CA, USA), according to the manufacturers’ instructions. The primary antibodies used in this study were mouse anti-CD5 monoclonal antibody (clone 4C7; Agilent), rabbit anti-CD117 polyclonal antibody (Nichirei BioScience, Tokyo, Japan), and rabbit anti-PRAME polyclonal antibody (cat. no. HPA045153; Sigma-Aldrich, St. Louis, MO, USA). The normal lymph node tissues were used as outer positive controls for CD5, gastrointestinal stromal tumours were used as controls for CD117, and normal testis tissues were used as controls for PRAME. When more than one core from the same patient showed immunoreactivity, we considered it to be a positive case. Differences in the classification rates of immunohistochemistry staining for PRAME between TSQCCs and thymoma or those between TSQCCs and healthy controls were evaluated using Fisher’s exact test. Results with *p* values of less than 0.05 were considered statistically significant. EZR (Saitama Medical Center, Jichi Medical University, Saitama, Japan), a modified version of R (the R Foundation for Statistical Computing, Vienna, Austria), was used for statistical analysis^41^.

### Comparison of *PRAME* mRNA expression in TSQCC, PRAME-negative thymoma, and PRAME-positive thymoma by digital mRNA counts

*PRAME* mRNA levels in TSQCCs (n = 10) and thymomas (n = 29), which were subdivided based on negativity (n = 21) or positivity (n = 8) for PRAME immunohistochemistry in tissue microarray, were analyzed and compared using the nCounter PanCancer Immune Profiling Panel (NanoString Technologies, Inc.). The methodology used for digital mRNA counts and data analysis in this comparative analysis is the same as that in comprehensive mRNA expression profiling by digital mRNA counts as described above.
